# Reduced Nucleotides, Thiols and O_2_ in Cellular Redox Balance: A Biochemist’s View

**DOI:** 10.3390/antiox11101877

**Published:** 2022-09-22

**Authors:** Lucien Bettendorff

**Affiliations:** Laboratory of Neurophysiology, GIGA Neurosciences, University of Liège, 4000 Liège, Belgium; l.bettendorff@uliege.be; Tel.: +32-4-366-5967

**Keywords:** glutathione, thiols, NADH, FADH_2_, oxidative stress, reactive oxygen species, mitochondria

## Abstract

In the present review, which is aimed at researchers, teachers and students in life sciences, we try to show how the physicochemical properties of the elements and molecules define the concept of redox balance. Living organism are open systems traversed by fluxes of energy and matter. During catabolic oxidative metabolism, matter—mostly hydrogenated organic molecules—is oxidized and ultimately released as CO_2_. Electrons are passed over to coupling molecules, such as NAD+ and FAD, whose reduced forms serve as electrons donors in anabolic reactions. Early photosynthetic activity led to the accumulation of O_2_ and the transformation of the reduction to an oxidizing atmosphere, favoring the development of oxidative metabolism in living organisms. We focus on the specific properties of O_2_ that provide the chemical energy for the combustion reactions occurring in living cells. We explain the concepts of redox potential and redox balance in complex systems such as living cells, we present the main redox couples involved in cellular redox balance and we discuss the chemical properties underlying their cellular roles and, in particular, their antioxidant properties in the defense against reactive oxygen species (ROS). Finally, we try to provide an integrative view emphasizing the interplay between metabolism, oxidative stress and metabolic compartmentation in mammalian cells.

## 1. Introduction

One of the characteristics of living beings is that they maintain an ordered state far from equilibrium by means of energy consumption. Simply put, living organisms are crossed by a flow of matter (carbon-containing molecules) transformed by their metabolic activity: phototrophs such as plants use the quantum energy of light to transform CO_2_ and H_2_O to organic molecules such as sugars, while animals use the energy stored in these organic molecules and dioxygen (O_2_) for all essential functions while transforming them back into CO_2_ and H_2_O. Most of the energy of animal cells comes from the oxidation of organic substrates by O_2_.

**Figure 1:**
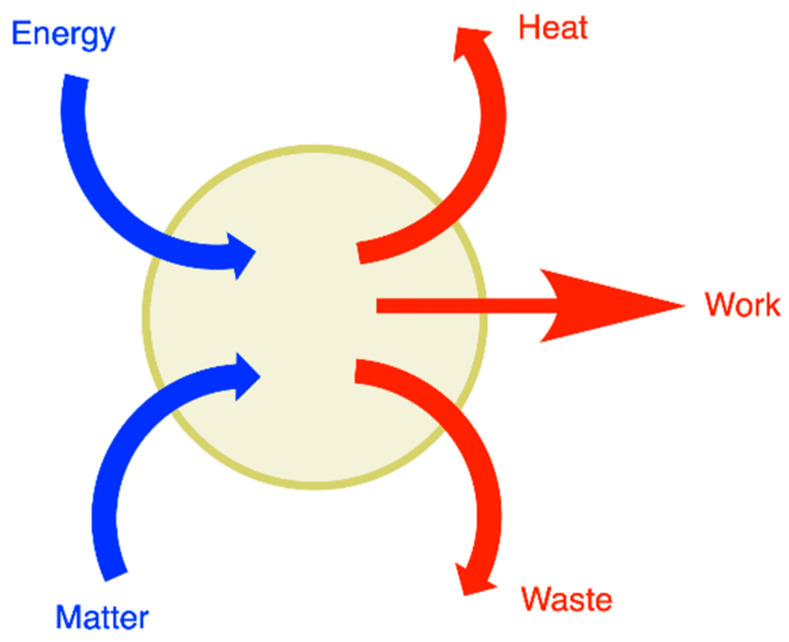
Living organisms are open thermodynamic systems traversed by energy and matter. The energy is dissipated as heat or converted to work. While some of these flows are vectorial (transport of a molecule across a membrane, heat transfer), others are not (chemical reactions).

During its path through living organisms, matter is transformed (glucose to CO_2_, for instance) while energy (chemical or electromagnetic) is converted into other forms such as movement or dissipated as heat (Figure 1). These processes—in fact, a network of interrelated and tightly controlled chemical reactions—are grouped under the term “metabolism”.

Hence, living organisms are traversed by flows of energy and matter, making them the paradigm of what thermodynamicists call an open system (Figure 1, [1]). Flows arise as a result of the existence of potential differences. It is essential for living organisms to maintain the potentials responsible for these flows. Indeed, the dissipation of these potentials leads to an equilibrium situation, meaning death. Hence, living organisms—and, in their most simple form, living cells—are nonequilibrium systems in a dynamic steady-state maintained at the expense of energy [2]. The whole set of regulatory mechanisms aimed at maintaining this steady state is called homeostasis. Here, we define a flux (*J*)—a vectorial transport driven by a force and corresponding to the movement (or flow) of a number (*n*) of a molecule X across a fixed area A—as a function of time $J\propto \frac{\mathrm{dn}}{\mathrm{dt}}$. When the flux is constant, we speak of a steady-state, and *n* is independent of time (dn/dt = 0).

It is not the aim of this essay to review the various mechanisms involved in maintaining the cellular redox balance (which have been excellently reviewed elsewhere; see, for instance, [3,4,5,6]); rather, we want to focus on the basic physico-chemical processes at the origin of the cellular redox potential and its relation to the general cellular metabolism and energy balance.

## 2. Free Energy Is the Driving Force for Maintaining Cell Dynamics

**Figure 2:**
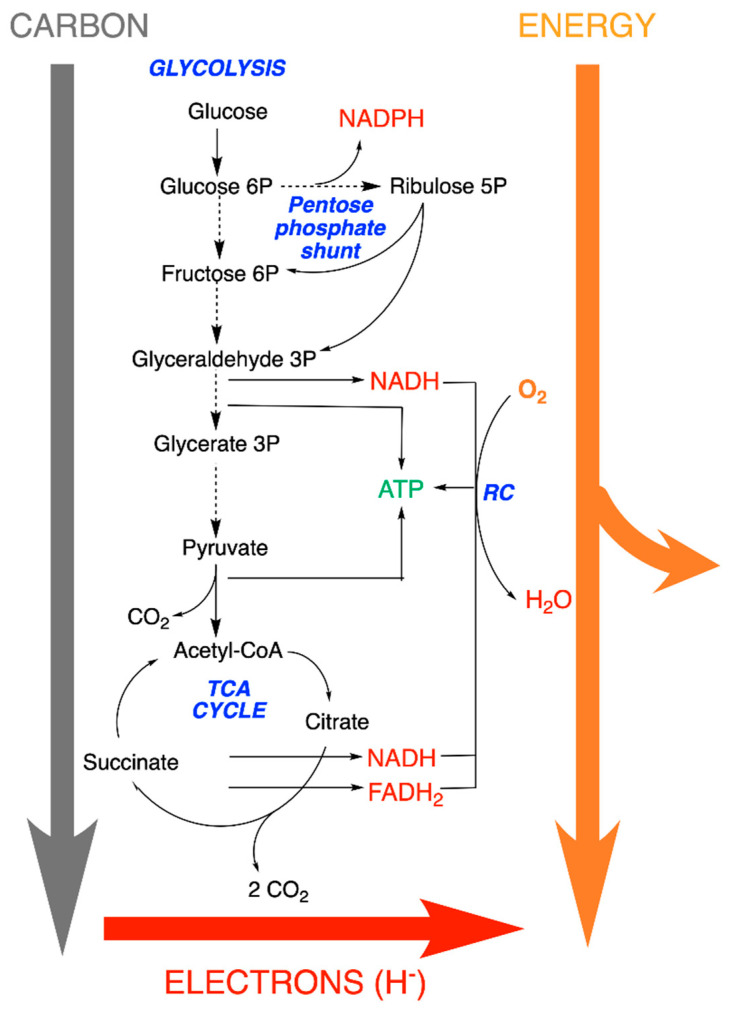
Catabolic flows of matter (carbon), energy and electrons through the central metabolic pathways (glycolysis, tricarboxylic acid (TCA) cycle and pentose phosphate shunt). For simplification, no subcellular compartmentation is indicated, and the scheme may concern any prokaryotic or eukaryotic organism. The driving forces depend on differences in concentrations and in the structural arrangement between products and reactants’ free energy (∆*G°*) and redox potential (∆*E*). (RC, respiratory chain).

We will essentially consider the catabolism of glucose through glycolysis, the tricarboxylic acid cycle (TCA) and the respiratory chain (RC), according to the thermodynamically irreversible global reaction (Figure 2):

C_6_H_12_O_6_ + 6 O_2_ → 6 CO_2_ + 6 H_2_O (1)

This metabolic activity leads to a flow of matter of mostly carbon-containing molecules, such as glucose, that are taken up by the cells and transformed; ultimately, the carbon is released as CO_2_. Hence, we must distinguish between true (vectorial) flows of matter (transport of glucose inside the cells or diffusion of CO_2_ outside the cells) and metabolic fluxes that consist in the transformation of matter.

The flux (*J*) of metabolites through a reaction is equal to the velocity of the forward reaction v_f_ minus the velocity of the reverse reaction v_r_ [7]:

*J* = v*f* − v*r*

This relationship holds true for enzyme-catalyzed reactions, which is the case for most metabolic reactions. In practice, the rate of metabolic pathways is controlled by rate-limiting steps that operate far from equilibrium (∆*G* << 0; see the reactions catalyzed by phosphofructokinase and pyruvate kinase in glycolysis) [8].

One of the major types of reactions in living cells involves the transfer of electrons from a donor (the reductant) to an acceptor (the oxidant), giving rise to a “flow” of electrons. However, in biological systems, electron flows are always local, electrons being only transferred over very short distances from one complex to another.

Typically, an electron donor is a molecule that gains stability by losing electrons, while the electron acceptor gains stability by accepting electrons. The regulation of these oxidation-reduction reactions—or, in short, “redox” reactions—is of particular importance for cell survival, and dysregulation of the so-called redox balance is either the origin or the consequence of many pathological conditions, leading to the production of cell-toxic byproducts—the so-called reactive oxygen species (ROS) [9,10].

We will not use the mathematical formalism of nonequilibrium thermodynamics to quantify the various flows, which is beyond the scope of this article (for those interested, see [11]). However, it is understood that the thermodynamic driving force (molar Gibbs free energy difference (∆G) or free energy dissipated by a reaction) for a reversible reaction is a function of the ratio of the forward over the reverse reaction fluxes [12,13]:

(2) $$∆G=-RT\ln\frac{J^{+}}{J^{-}}$$

Fluxes are complex nonlinear functions of concentrations, but for simplification, we will assume that the fluxes are constant (steady-state conditions maintained by homeostasis), which allows us to directly link the fluxes to the standard molar Gibbs free energy depending on the steady state reaction quotient (*Q*) representing the real concentrations of reactants and the redox potential in the case of redox reactions (Table 1).

Put differently, a flow of matter arises from the existence of differences in concentrations (Q) and differences in the structural arrangement between initial and final reactants (∆G°), and a flow of electrons arises from differences in redox potential (∆E).

Bond-formation between elements in their standard state to form molecules is accompanied by a decrease in energy. Hence, bond-formation enthalpies are always negative, and the more negative they are, the stronger the bond. The carbonated end-product (CO_2_) is at a higher state of oxidation than initial reactants: CO_2_ is the most oxidized form of carbon (oxidation state + 4), while organic molecules are hydrogenated and, hence, in a more reduced state than CO_2_. During the metabolic activity, the electrons (generally under the hydride form H^−^) are transferred to NADPH and NADH. The latter is oxidized in the respiratory chain by O_2_ and the electrons transferred to H_2_O (Figure 2). Just as some reactions are coupled to ATP synthesis, many redox reactions are coupled to the synthesis of reducing equivalents (NADH and FADH_2_). It is important to recall that no compound can lose an electron without an acceptor.

This catabolic activity is only possible because of a difference in free energy between the reactants and the products. Molecules with weaker bonds (high-energy bonds) are replaced by molecules with stronger bonds (low-energy bonds). This holds true only when the number of electron-pair bonds (a double bond counts for two bonds) remains unchanged [16]. Part of the free energy is stored in ATP used for anabolic activities, and another part is dissipated into the environment as heat.

What holds for metabolic sequences can be extrapolated to whole living organisms. Indeed, among the different lifeforms, some lifeforms called phototrophs directly use solar energy. They transform solar energy into chemical energy and synthesize different forms of organic molecules (starch, for example) and O_2_ (Table 2). Organisms that live off these latter molecules and break them down via a series of redox reactions (involving O_2_) are called chemotrophs. At the level of an ecosystem, there is therefore a macroscopic coupling between the organisms that synthesize organic molecules and the others that use them.

Energy coupling is a fundamental concept in biochemistry. Exergonic reactions (such as most catabolic reactions) are coupled to endergonic reactions (most anabolic reactions), so the global standard free energy ∆*G°* (or ∆*G*—actual concentrations of the reactants) is negative.

## 3. Redox Potential and the Chemical Basis for the Role of O_2_ as an “Energy-Rich” Molecule

Chemistry can be defined as the science studying the electron clouds of atoms (in opposition to physics, which studies the nucleus) and the reactions involved. To some extent, all chemical reactions involve an exchange of electrons between chemical partners, but true redox reactions can be recognized by the fact that the reaction occurs even when the molecular species are physically separated in different compartments or half-cells for experimental purposes, provided that these compartments are connected by an electrical circuit [19].

While the importance of redox balance and oxidative stress is a relatively new notion, going back to the 1980s [20], the notion of redox potential goes back to the work of the English chemist Joseph Priestley and the French chemist Antoine-Laurent Lavoisier in the 18th century and was further developed by electrochemists in the 19th century [19]. Historically, electrochemistry is related to a branch of chemistry studying the electrical currents generated by chemical reactions.

**Figure 3:**
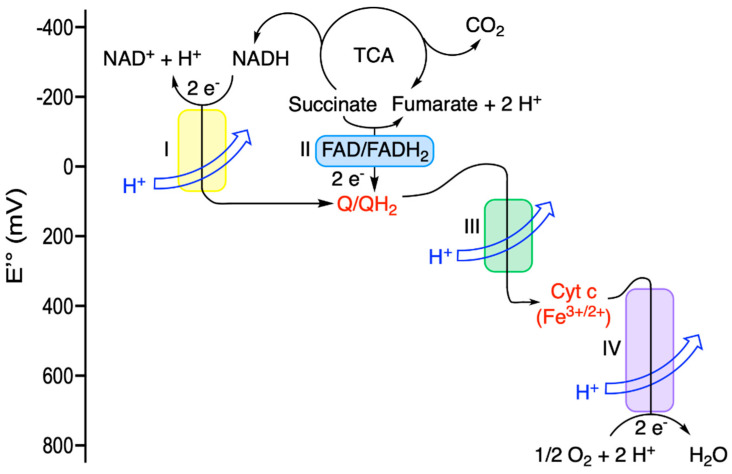
Electron flow from NADH and FADH_2_ to O_2_ in the mitochondrial respiratory chain. While the first reactions involve the transfer of H^−^ + H^+^ to the acceptor Q, complexes III and IV act as electron wires coupled to proton pumps.

Charges move between regions of different electrostatic potentials. Hence, the closing of an electrical circuit will trigger a flow of electrons (current) from a region of high electrostatic potential (the negative electrode) to a region of low electrostatic potential (the positive electrode). In metals, which have a high electrical conductivity, electrons can move relatively freely. This is not the case in solutions. There are no such thing as stable free electrons in an aqueous solution, and electrons are transferred from a donor molecule to an acceptor molecule either directly or indirectly through enzymes. In this case, both the donor and acceptor are assigned an empirically designed physical quantity termed oxidation-reduction or redox potential (E, expressed in volt) (Figure 3, Table 3).

It is impossible to measure the absolute redox potential. Hence, the redox potential is a relative quantity and is defined as a measure of the tendency of a molecule to donate electrons to a reference acceptor molecule by convention H^+^ or, on the other hand, to accept electrons from the reference donor H_2_, yielding the standard redox potential (E°) defined at pH 0. As physiological reactions most often happen close to pH 7, biochemists use a standard apparent redox potential (∆E′°) at pH 7 [21]. According to biochemical conventions (pH 7 versus pH 0 for the chemical convention), the E′° of H^+^/H_2_ is not 0 but −0.42 V (Table 3).

In the major metabolic pathways, redox reactions involve carbon-containing molecules. When carbon atoms undergo changes in the redox state, electrons are moved in combination with hydrogen atoms (see, for instance, the oxidation of NADH, FADH_2_ or reduced glutathione in Table 3)—hence, the denomination of “dehydrogenases” for the enzymes catalyzing such reactions. The sole movement of electrons is generally limited to the reduction of metals, mostly Cu^2+^ and Fe^3+^, the latter very often found in iron-sulfur clusters or hemes (cytochromes, for instance; Figure 3) within protein complexes. Indeed, electrons only transfer energy when they are moving through a “wire”, which, in the case of the respiratory chain complexes, fuels H^+^ transport towards the inner compartment (matrix) by respiratory complexes I, III and IV. However, when the chemical bonding changes (for instance, in the reaction Q + 2H^+^ + 2e → QH_2_; Table 3, Figure 3), differences in bond energies between products and reactants drive the electron flow. As such, QH_2_ can be considered of a lower energy (more negative) than Q: QH_2_ carries electrons, while Q carries energy [16].

In contrast to what is sometimes implied, the reduced form of a molecule is not necessarily the most “energy-rich”. As detailed by Schmidt-Rohr, the half-reaction for the oxidation of Fe^2+^ in cytochrome c (Fe^2+^_(cytc)_ + e → Fe^3+^_(cytc)_; Table 3, Figure 3) is actually energetically uphill, as it corresponds to an ionization [16].

In complex IV, four electrons are transferred stepwise to one molecule of O_2_ for complete reduction to form two molecules of H_2_O; the four protons play the role of Brønsted acids [22].

As illustrated in Figure 2 and Figure 3, the oxygen atoms of O_2_ consumed by the respiratory chain end up in H_2_O, while the carbon and oxygen atoms of glucose are released as CO_2_. Hence, the metabolisms of carbon and O_2_ are physically separated but connected through the intermediate NAD^+^/NADH couple.

As an example of a physiological redox reaction, we will consider the oxidation of NADH by O_2_ catalyzed by the mitochondrial respiratory chain (Figure 3):

1/2 O_2_ + NADH + H^+^ ⇆ H_2_O + NAD^+^    ∆E′° = +1.14 V (3)

The redox potential for each of the two half-reactions is (Table 3):

1/2 O_2_ + 2 H^+^ + 2 e^−^ → H_2_O      E′° = + 0.82 V

NAD^+^ + 2 H^+^ + 2 e^−^ → NADH + H^+^     E′° = −0.32 V

The change in free energy associated with the global reaction is

∆G′° = −z F ∆E′° = −2 × 96.5 × 1.14 = −220 kJ/mol(4)

where F is the Faraday constant and *z* the number of electrons transferred.

As O_2_ is the universal electron acceptor in all aerobic organisms, it is important to understand the chemical reason for the high oxidative capacity of O_2_.

According to Schmidt-Rohr, the enthalpy change of combustion *(∆_c_H°*) can be calculated from the bond-dissociation energies (*D_i,m_*) [24]:

(5) $$∆H_{c}^{{}^{\circ}}=\underset{i\;\left(\mathrm{reactants}\right)}{\sum }v_{i\;}\underset{m}{\sum }D_{i,m\;}-\underset{i\;\left(\mathrm{products}\right)}{\sum }v_{i\;}\underset{m}{\sum }D_{i,m\;}$$

The sum over i considers all reactants and products, while the sum over m represents all bonds within reactants and products.

Consider the global reaction for the combustion of glucose by O_2_ (reaction I):

C_6_H_12_O_6_ + 6 O_2_ → 6 CO_2_ + 6 H_2_O     ∆H°_c_ = −2805 kJ/mol

In this fundamental biochemical reaction sequence, the total number of electron-pair bonds (counting the double bonds as two) is the same in the reactants (36) and products (36): only the way the atoms are arranged differs.

**Figure 4:**
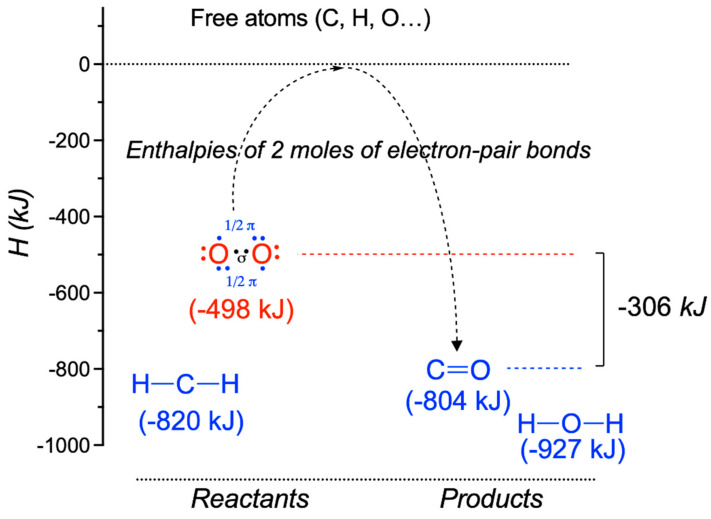
Bond-formation enthalpies of two moles of electron-pair bonds in O_2_ and CO_2_. The bond formation enthalpies are lower (more negative) for stronger bonds [24]. O_2_ is represented in the ground state of the triplet diradical dioxygen molecule with the two unpaired electrons participating in the π bonding. π electrons are shown in blue, while σ electrons are in black (modified from [24]).

As the bond formation enthalpy (∆H°_f_) is always negative, the replacement of a weaker bond (∆H°_f_ less negative) by a stronger bond (∆H°_f_ more negative) always releases energy. This concept can be generalized to any reaction, provided the number of electron bonds remains unchanged (which is generally the case in biochemical reactions). This point has been thoroughly analyzed by K. Schmidt-Rohr [24] in the case of combustion reactions, and the author points out that O = O has a much less negative bond-formation energy (−498 kJ/mol) than the C = O bond in CO_2_ (−804 kJ/mol), as well as in other pairs of formal bonds. He shows that this difference of −306 kJ/mol accounts for the major part of ∆H°_c_, making it the driving force for all combustion reactions involving O_2_ (the majority) as an oxidant (Figure 4). In other words, the double bond in O = O is weaker than the double bonds in other molecules; the difference makes the combustion reaction highly exothermic [24,25,26]. Accordingly, O_2_ provides about 75% of the energy of combustion, while the organic part contributes only 25% [24]. Hence, the value of hydrocarbons would be that they unlock the energy stored in O_2_ [16].

Schmidt-Rohr therefore labels O_2_ an “energy-rich” molecule, as, during a combustion reaction, the substitution of the O = O double bond by a C = O double bond is always accompanied by a release of energy. According to this view, the energy released in combustion reactions comes from the substitution of the weak O_2_ double bond by a stronger one and not from the particular “energy richness” of the reduced substrates (sugars, lipids, etc.) [24]. In favor of this view is the fact that the break-down of reduced molecules by pathways other than combustion does not release a significant amount of energy [16].

The exceptional properties of O_2_ have recently been stressed by Borden et al. [27]. As a triplet diradical (Figure 4), O_2_ would be expected to be kinetically unstable. However, though highly abundant in our atmosphere, O_2_ is unreactive against other molecules in reactions that are thermodynamically exothermic (hydrogen abstraction, for instance). This situation is most probably due to the resonance stabilization of the strong π bond (418 kJ/mol). On the other hand, the σ bond is relatively weak (19 kcal/mol or 80 kJ/mol, footnote 57 in [27]), allowing for the formation of two molecules of H_2_O and the release of a large amount of energy [27]. This contrasts with most organic molecules, where the π bond is weaker than the σ bond. 

In other words, the weak σ bond is responsible for the thermodynamic instability of O_2_, while the strong π bond increases the activation energy for the reaction with other molecules.

As mentioned above, O_2_ is a very powerful oxidant capable of oxidizing just about every other molecule (reductant) present in the cells (Table 3), and cells take advantage of this property for respiration. O_2_ must be continuously regenerated by photosynthetic organisms, and what is an advantage for animal cells becomes a major obstacle for photosynthetic organisms that produce O_2_ by photolysis:

2 H_2_O + 4 photons (light) → 4 e^−^ + 4 H^+^ + O_2_ (6)

The highly unfavorable energetics of this reaction are, however, compensated by (1) the production, at the expense of quantum energy (*hv*), of an oxidant (P680^+^; Table 3) so powerful and high in energy that it is able to extract electrons from water and release O_2_, a process called photolysis, and (2) the high availability of H_2_O for photolysis.

It is thought that photosynthesis is at the origin of the accumulation of O_2_ in the terrestrial atmosphere, and its switch from a reductive to an oxidative state approximately two billion years ago is known as the Great Oxygenation Event, triggering the first massive extinction in the planet’s history [28,29,30]. Indeed, O_2_ may capture one or two electrons generating ROS, such as superoxide (O_2_^−^) and hydroxyl (OH) radicals and non-radical peroxides (H_2_O_2_). ROS are highly toxic by inactivating metabolic enzymes, and only those organisms capable of developing highly efficient defense systems against ROS (in particular, ROS-neutralizing enzymes such as superoxide dismutase, peroxidases and catalase) were able to survive.

An important consequence of the Great Oxygenation Event was that O_2_ became available for the combustion of organic substrates (instead of fermentation), boosting the amount of free energy capable of being extracted and allowing for the evolution towards more complex organisms such as animals [28].

## 4. The Special Role of Hydrogen in Biochemical Redox Reactions

As already mentioned above, in organic chemistry, the reduction of a carbon atom is generally concomitant with the gain of a hydrogen atom, i.e., it gains a bond to the less electronegative hydrogen, leading to a stepwise transformation of functional groups during reduction: 
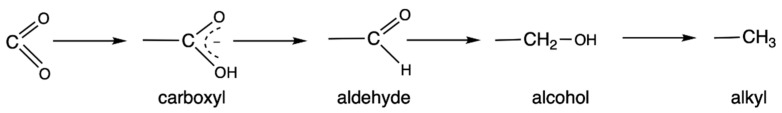


In this case, the reduction of the carbon atom is also concomitant with a decrease in the number of C–O bonds. 

The standard redox potentials of molecules are strongly affected by their bond energies: weak bonding (i.e., high chemical energy) of the oxidized form of the molecule lowers the standard reduction potential.

The reduced coenzymes NAD(P)H and FADH_2_ (keep in mind that it is carbon atoms that are reduced) perfectly illustrate the importance of hydrogen in biological redox reactions. Hydrogen is a very particular element, as it can be transferred from one molecule to another either as H^+^ (a cation, in acid/base reactions), as H^•^ (in homolytic isomerization reactions with cobalamin as a coenzyme) or as H^−^ (hydride anion in redox reactions [31,32]). The latter can be catalytically extracted from a molecule of the type HO–C(R_2_)–H by NAD^+^—for instance, with the concomitant oxidation of the carbon atom: 
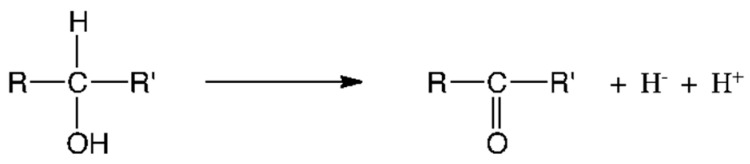


Schmidt-Rohr even postulates an equivalence between NAD(P)H + H^+^ and H_2_ [16], the latter being able to replace the former in many reactions with a similar energetic outcome. NAD(P)H and FADH_2_ have relatively close reduction potentials (Table 3). In that sense, these molecules could be considered as biological analogues of H_2_ [16].

Hydride transfer (H^−^, or, the equivalent of two electrons and H^+^) is an important reaction in many biological processes—in particular, those using NAD(P)H, FADH_2_ or QH_2_ as reductants. The mechanism of such reactions—the one-step transfer of H^—^ transferred or the three-step “electron–H^+^–electron” transfer—remains disputed. Experimental evidence on the electron transfer from NADPH to protochlorophyllide would suggest the latter [33].

**Figure 5:**
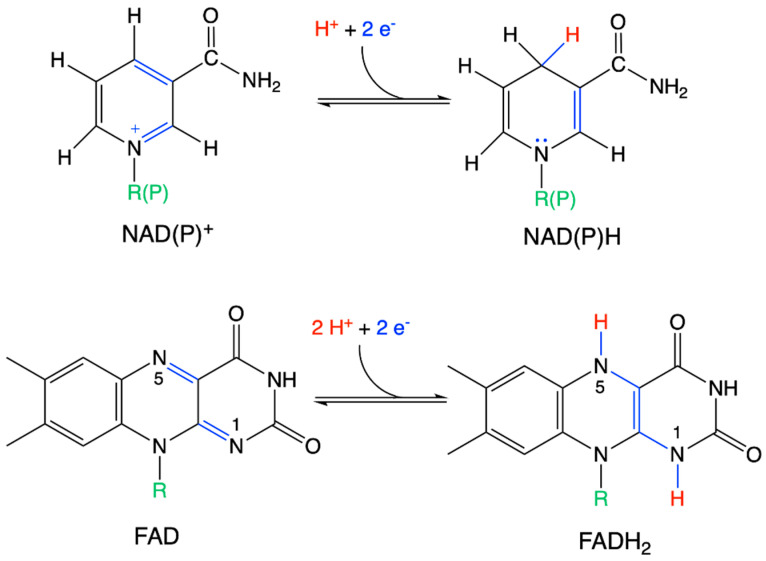
Structures of the oxidized and reduced forms of NAD(P)^+^/NAD(P)H and FAD/FADH_2_. Electron rearrangements are shown in blue and hydrogenations are shown in red.

In the reduced NADH (or NADPH) molecule, the pyridine ring loses its aromatic character and is hence eager to lose the extra two electrons to return to its aromatic state, bringing extra stabilization (Figure 5). In the FAD/FADH_2_ couple, reduction leads to the loss of the resonance energy of the trans butadiene frame [31]. In each case, changes in the redox state are accompanied by an instant reorganization of molecular orbitals in such a way that the oxidized form is associated with a free orbital of particularly low energy, while the reduced form is associated with an occupied orbital of particularly high energy. Therefore, these molecules have a natural tendency for the oxidized form to accept electrons and for the reduced form to donate electrons [32].

**Figure 6:**
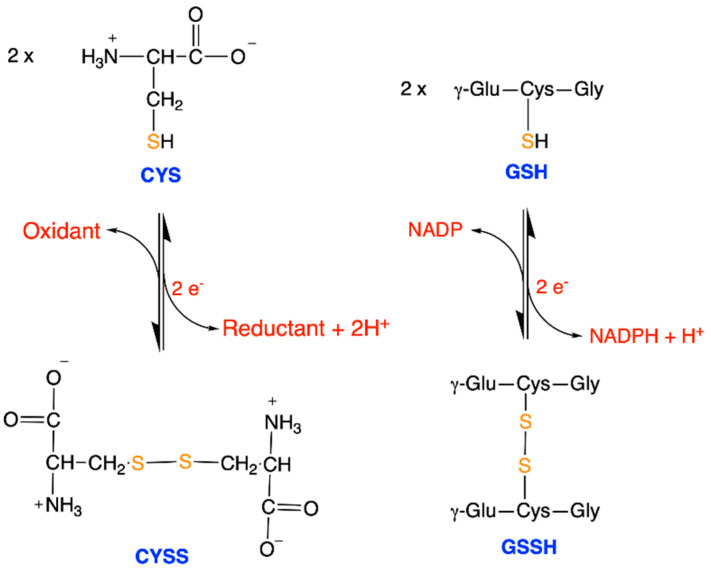
Structures of cysteine (CYS)/cystine (CYSS) and reduced glutathione (GSH)/oxidized glutathione (GSSG). Both reactions are very similar, consist in the formation of a disulfide bond from cysteine thiols and involve the transfer of two electrons and 2 H^+^ (or H^−^ and H^+^, depending on the molecular mechanisms involved).

Other examples of reduction are the saturation of a double bond between two aliphatic carbon atoms (R_2_C = CR_2_ → R_2_CH—CHR_2_) or the formation of thiols from disulfides (Figure 6). Once again, these reactions operate by hydride transfer (H^−^ + H^+^).

## 5. Metabolic Blocks and Metabolic Compartmentation of Redox Couples

**Figure 7:**
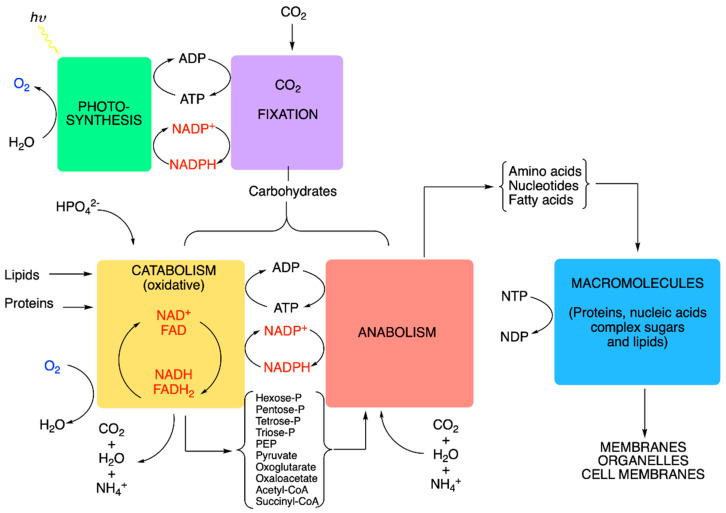
Metabolic compartmentation of redox couples (adapted and modified from [34]). Schematic representation of intermediary metabolism. In heterotrophic organisms, there are three metabolic blocks (catabolism, anabolism and macromolecule synthesis), to which two other blocks are added (photosynthesis, CO_2_ fixation) in autotrophs. Notice the importance of five inorganic molecules: H_2_O, O_2_, CO_2_, NH_4_^+^ and HPO_4_^2−^; the latter are the N and P donors. (PEP, phosphoenolpyruvate).

In an idealized cell, we can distinguish five metabolic blocks: photosynthesis (light phase), CO_2_ fixation (dark phase), catabolism, anabolism and the synthesis of macromolecules (Figure 7). The first two are only present in photosynthetic organisms, while the latter three exist in all living cells, both photosynthetic and heterotrophic.

All blocks are closely interconnected by various coupling agents. We can distinguish three categories of coupling agents:

(1) Nucleoside triphosphates with a high phosphate group transfer potential—in particular, the ATP/ADP couple.
(2) The redox coupling agents are NAD^+^/NADH, NADP^+^/NADPH and FAD/FADH_2_.
(3) Approximately 10 organic compounds that constitute products of the catabolic block and are used as building blocks for the anabolic block. Among these are two thioesters (acetyl-CoA and succinyl-CoA, with a high acetyl transfer group potential), three 2-oxoacids (pyruvate, oxaloacetate and oxoglutarate), phosphoenolpyruvate and four phosphorylated sugars (from C3 to C6).

NADH is produced by the catabolic pathways of glycolysis (cytoplasm), the ß-oxidation of fatty acids (mitochondria) and the TCA cycle (mitochondria), and its reducing equivalents are mainly used by the mitochondrial respiratory chain. The NAD^+^/NADH couple is thus essentially restricted to the catabolic block (Figure 7).

NADPH is produced in the oxidative part of the pentose phosphate shunt (cytoplasm) and in plants during photosynthesis. NADPH is the major electron donor for the reductive biosynthesis of fatty acids, amino acids and nucleic acids in the anabolic block. It is also essential for regenerating GSH, a powerful antioxidant, protecting against free radicals.

NADH and NADPH are therefore not metabolically interchangeable, because the dehydrogenases involved in the oxidative and reductive pathways are highly specific for their respective coenzymes. This specificity is due to the presence or absence of phosphate in the 2′ position of ribose. In the cytoplasm, cells maintain a low [NADH]/[NAD^+^] ratio (<0.02), promoting the oxidation of metabolites. On the other hand, the ratio [NADPH]/[NADP^+^] is high, promoting reductive biosynthesis.

It is thus evident that the main redox couples are intimately linked to the cell energy metabolism (Figure 7), and imbalances in the cellular redox state will have major consequences for cellular energetics and the metabolism, leading to a variety of pathological conditions [35]. Indeed, glucose hypometabolism as well as oxidative stress are among the earliest changes observed on the path towards cognitive impairment in Alzheimer’s disease, for instance [36,37].

## 6. Extrapolation of the Concept of Redox Potential to Complex Systems: The Redox Environment

As mentioned above, the redox potential is defined under very specific (laboratory) conditions for two half-reactions. In complex systems, be it the planetary atmosphere or living cells, many reductants and oxidants coexist within a single compartment. Hence, the “redox state” of such complex systems will be the weighted mean of all half-reactions present.

Expressions such as reducing or oxidizing atmosphere are common in the literature—for instance, in relation to the evolution of Earth’s atmosphere. It is, however, less clear what it means. Schafer and Buettner [38] proposed a definition of the redox environment that they link to the number of electrons available. Quantitatively, the redox environment of a medium can be represented by a reduction potential (*E_i_* or *E_i_′*, expressed in terms of voltage and calculated by the Nernst equation) whose capacity corresponds to the total stored charge or the total number of electrons available:

(7) $$\mathrm{Redox}\;\mathrm{environment}=\sum_{i=1}^{n\;\left(couple\right)}E_{i}\;\times \;{\left[\mathrm{reduced}\;\mathrm{species}\right]}_{I}$$

Hence, in a reducing atmosphere, the main molecules—such as, for instance, dihydrogen, methane, nitrogen, disulfides, ammonia and carbon dioxide—would be good building blocks for more complex organic molecules, but they would not be very efficient from an energetic point of view (low O_2_ concentrations). The earliest living organisms were probably anaerobic autotrophs, extracting energy from reactive nitrogen or sulfur compounds or, alternatively, methanogens [39,40].

However, in an environment rich in oxidants (present atmosphere) containing 21% of O_2_, the latter, with its very low standard redox potential (0.82 V; Table 3), and the energy-rich nature of its double bond will be reduced by practically all electron donors. As the standard redox potential can be linked to the standard free energy by Equation (4) ∆G′° = −*z* F ∆E′° [14], oxidations will be energetically favored in such an atmosphere. On the other hand, reducing reactions are energetically unfavorable.

With this respect, it must be emphasized that the physiological redox potential of a redox couple depends on the relative concentration of the oxidized and reduced species present in addition to the standard redox potential [14]:

(8) $$E^{\prime }=E^{\prime }{}^{\circ}+\frac{RT}{zF}\;\ln\;\frac{a_{ox}}{a_{red}}$$

where a is the activity (or the effective concentration) of the reduced and the oxidized species of the couple, respectively, *R* is the gas constant, *T* is the absolute temperature, *F* is the Faraday constant and *z* is the number of electrons transferred. This means that unfavorable energetics can, to some extent, be overcome by increasing the $\frac{a_{ox}}{a_{red}}$ ratio. Note that, in dilute solutions, the activity *a* can be replaced by a concentration term.

As emphasized by Schafer and Buettner [38], it may be difficult to know all the redox couples in a cellular compartment and even more difficult to measure their reduction potentials and concentrations. Therefore, it is more convenient to identify a redox couple representative of a given cellular compartment.

The couple formed by reduced glutathione/oxidized glutathione (GSH/GSSG) is a representative redox buffer of the cytoplasmic compartment. Thiols (–SH) represent the most reduced form of sulfur in biomolecules and are mainly found in cysteine [41]. Thiols have a p*K*_a_ of 8.0–8.5, depending on the molecular environment. Therefore, at physiological pH, a small proportion of cysteines is susceptible to deprotonation, yielding the highly reactive thiolate anion (-S^−^) [42,43].

In the case of the CYS/CYSS and GSH/GSSH couples, the number of products and the number of substrates are unequal; hence, the redox potential also depends on the absolute concentrations, in addition to their ratios [14]:

$$E^{\prime }=E^{\prime }{}^{\circ}+\frac{RT}{zF}\;\ln\;\frac{\left[\mathrm{GSSG}\right]}{\;{\left[\mathrm{GSH}\right]}^{2}}$$

This explains why the total glutathione pool size (defined by [GSH] + 2[GSSG]) must be maintained at relatively high concentrations (typically around 10 mM) in order to fulfill its role as a redox buffer [14]. The same argument also holds for the CYS/CYSS couple.

GSSH and CYSS are symmetrical dimers. Recent studies described the post-translational modification of protein cysteines by S-glutathionylation according to the reaction Protein-SH + GSSG ⇆ Protein-CyS-SG + GSH, yielding an asymmetrical derivative [44,45,46]. Protein S-glutathionylation might be involved in oxidative stress, the protection of protein cysteine residues and cell signaling.

Under conditions that are still poorly defined, the thiamine molecule may also undergo thiazole ring opening, yielding a thiol derivative that might be involved in thiol–disulfide exchange reactions with presently unknown partners [47].

## 7. Cellular Compartmentation of Redox Balance

**Figure 8:**
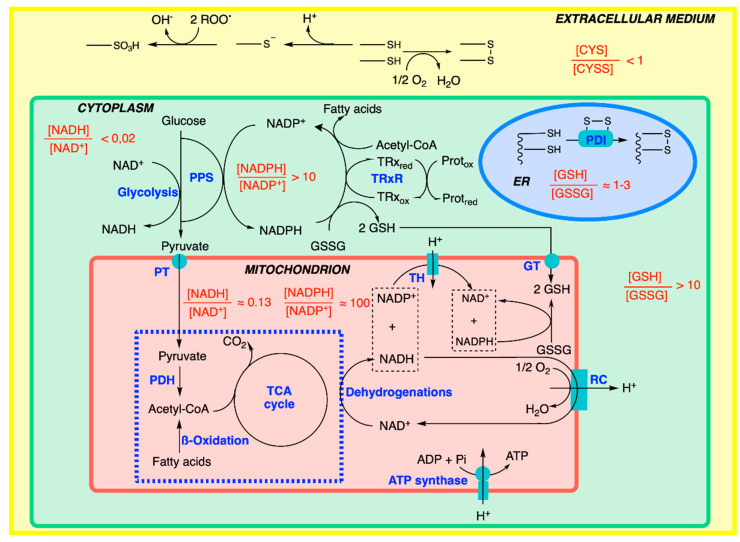
Redox and thiol balance in an animal cell. For explanations, see the text. RC, respiratory chain; GT, GSH carrier; PDH, pyruvate dehydrogenase complex; PDI, protein disulfide isomerase; PT, pyruvate transporter; ER, endoplasmic reticulum; PPS, pentose phosphate shunt; TH, H^+^-dependent transhydrogenase; TRx, thioredoxin; TRxR, thioredoxin reductase.

Electrons are transferred from organic molecules (sugars, fatty and amino acids) to intermediates (NAD^+^/NADH) and then to oxygen (O_2_/H_2_O), which promotes the maintenance of a reducing medium in the cells (Figure 8). These metabolic sequences involve different cellular compartments, the main ones being the cytoplasm and the mitochondrial matrix.

The redox potential varies among the different cellular compartments: it is highest, far from equilibrium, in the mitochondrial matrix, followed by the cytoplasm, and it is lowest in the endoplasmic reticulum (ER) [42]. Available data in organelles are limited: while the nucleus is relatively reducing, the lysosomes and secretory vesicles are oxidizing [48,49].

In terms of capacity, the main intracellular redox pairs are NADH/NAD^+^, NADPH/NADP^+^ and reduced glutathione/oxidized glutathione (GSH/GSSG).

One of the main ROS scavenging molecules is reduced glutathione (a thiol), which is also the main effector responsible for maintaining the intracellular redox state of the cells. Thiols are highly nucleophilic, making them a target of choice for the highly electrophilic ROS [42]. Thiols, in the form of cysteine residues, are present in most proteins, and their oxidation by ROS leads to alterations in protein structure and function. It is therefore not surprising that thiols (in reduced glutathione and cysteine) were selected by evolution to become the major actors by competitively protecting cells against oxidative damage, leading to the concept of thiol redox switch [50,51,52].

The concentration of GSH in cells varies between 1 and 10 mM. It alone makes up the bulk of the cell’s free thiols and provides a large pool of reducing equivalents (Figure 8). The GSH/GSSG pair can be considered as a redox buffer and is, as such, an indicator of the redox potential of the cell (see above).

In the cytoplasm and mitochondria, the ratio [GSH]/[GSSG] is greater than 10 [53]. This ratio is maintained at the expense of NADPH, produced in the oxidative part of the pentose phosphate shunt. In mitochondria, a particularly high ratio [NADPH]/[NADP^+^] (>100) is maintained at the expense of NADH thanks to an H^+^-dependent transhydrogenase (EC 1.6.1.1) catalyzing the reaction [54,55]:

H^+^_out_ + NADP^+^ + NADH ⇆ H^+^_in_ + NADPH + NAD^+^

In this case, the energy of the proton gradient is used to shift the equilibrium towards the synthesis of NADPH: NADH + NADP^+^ → NAD^+^ + NADPH. NADPH regenerates the GSH essential for defense against free radicals generated by the respiratory chain. GSH cannot be synthesized de novo inside the mitochondria and must be imported via transporters located in the inner membrane of the mitochondria. 

The high redox state of the cytoplasm and the mitochondrial matrix precludes the spontaneous formation of disulfide bridges. The thiol/disulfide balance is also regulated by a small 12 kDa oxidoreductase, thioredoxin (Figure 8), which is present in practically all living cells, and loss-of-function mutations are lethal in the developing embryo [56]. Thioredoxin contains two cysteine residues separated by two hydrophobic amino acids that may switch between the reduced thiol and oxidized disulfide forms. In its reduced state, it helps to keep target proteins (ribonuclease, insulin and many other proteins) in their reduced state. Reduced thioredoxin is regenerated in two steps by the flavoprotein thioredoxin reductase (EC 1.8.4.16) and NADPH [57,58,59]. Hence thioredoxin acts as a catalyzer, present in only micromolar concentrations in the cytoplasm [41].

In the lumen of the endoplasmic reticulum, the conditions are more oxidizing (the ratio [GSH]/[GSSG] is close to 1; [48,60]) than they are in the cytoplasm and mitochondria, facilitating the formation of disulfide bridges in proteins catalyzed by a protein disulfide isomerase (EC 5.3.4.1) [61,62]. The oxidized isomerase is regenerated by a complex enzymatic reaction involving molecular oxygen.

The extracellular medium is oxidizing if O_2_ is supplied to it (by arterial blood): the main redox couple here is the cysteine/cystine couple (CYS/CYSS) with a ratio of less than 1; the environment is therefore oxidizing [41,63]. The free thiols are oxidized spontaneously, either by the formation of disulfides by the reaction with oxygen or by the oxidation to sulfonates by the reaction with peroxides. For this reason, the thiols of the secreted proteins are generally protected by the formation of inter- and intramolecular disulfide bridges increasing the stability of the complexes (see the examples of keratin and collagen).

## 8. Antioxidant Defense Systems

In modern living organisms, imbalances in the redox state (either due to external or internal factors) can lead to the production of ROS, creating a state of oxidative stress leading to entropy production and extensive cell damage (breaks in DNA strands, lipid peroxidation, protein oxidation) [64]. Such mechanisms occur in normal aging but also in many pathological conditions such as Parkinson’s disease, Alzheimer’s disease, atherosclerosis, depression, inflammation, high-calory diet, cancer or even in the susceptibility to SARS-CoV-2 infection [65,66,67,68,69,70].

ROS are mostly byproducts of enzymatic reactions in various cellular compartments. The mitochondrial respiratory chain is the major source of ROS production in the cell [71]. Other sources are mitochondrial enzyme complexes such as 2-oxoglutarate dehydrogenase [72,73], the endoplasmic reticulum, peroxisomes, NADPH oxidases, nitric oxide synthase and many other enzymes [5].

It is important to understand that there are many forms of oxidative stress depending on the causative agent (radiation- or nutrition-induced, for instance) and the antioxidant defense systems [20]. Cells have developed various mechanisms to neutralize ROS and ROS formation.

Whilst there are many kinds of antioxidant defense systems in living cells, such as regulators of the expression of genes involved in the cellular defense against ROS or enzymes (catalase, superoxide dismutase or peroxidases), the term antioxidant should be limited to molecules (generally organic) that either slow down the oxidation of cellular components or eliminate ROS. Antioxidants eliminate oxidants by capturing electrons and get reduced in the same process. This can be either directly or indirectly by interrupting free radical chain reactions [74]. Potent antioxidants are thiols (glutathione), coenzyme Q, lipoate, vitamins A, C, D and E as well as many molecules of plant origin (flavonoids, polyphenols, etc.) [75].

In addition to the above-mentioned classical mechanisms regulating redox homeostasis, it becomes clear that other mechanisms are involved. Most of the genes involved in redox homeostasis (NADPH-dependent enzymes, thioredoxin, thioredoxin reductase, etc.) are targets for micro RNAs inhibiting their expression at the mRNA level, adding an additional degree of complexity in the cellular responses to oxidative stress [76].

Low levels of ROS are produced under physiological conditions and are involved in normal cellular functions such as redox signaling and in inflammatory processes [52]. The animal immune system, for instance, produces ROS to neutralize invading microorganisms.

It is well documented that the leaning of the redox balance towards pro-oxidants will lead to oxidative stress and disease. ROS can thus be considered as Janus molecules presenting both beneficial and toxic effects. This led some authors to classify oxidative stress into good stress (eustress) and bad stress (distress) [10,77,78].

Much less is known about the opposite phenomenon, namely, reductive stress, or, an imbalance of antioxidants over pro-oxidants and excess reducing equivalents [35]. Reductive stress affects cellular metabolism as it decreases ROS signaling function and may also interfere with proper protein folding in the ER, as protein disulfide formation requires an oxidizing environment [79]. Furthermore, reductive stress has been linked to diabetes [35,80].

## 9. Conclusions

By starting with the properties of some elements (carbon, hydrogen and oxygen), we have tried to provide an integrative biochemical picture of redox processes in animal cells. We voluntarily remained at a very molecular level, and our aim was to explain some basic concepts—such as the redox environment, the role of O_2_ as a high-energy molecule and the role of thiols in cellular redox compartmentation—that are often taken for granted and used without proper definition. Understanding these concepts is essential for developing new strategies and new antioxidants for the defense against ROS.

## Figures and Tables

**Table 1:** Driving forces for biochemical reactions in living cells.

Driving Force (J.mol^−1^)	Source of Driving Force
∆*G* = ∆*G°* + *RT* ln *Q*	Differences in the structural arrangement between initial and final reactants (∆*G°*) and concentrations (*Q*)
∆G=−z*F*∆*E*	$\mathrm{Differences}\;\mathrm{in}\;\mathrm{redox}\;\mathrm{potential}\;(∆E)\;(∆E=∆E+\frac{RT\;}{zF}$ln *Q*)

*Q* is the reaction quotient (dimensionless) and *z* the number of electrons transferred (dimensionless). *F* is the Faraday constant and ∆*E°* is the difference in the standard redox potential between the donor and acceptor redox couples [14,15].

**Table 2:** Classification of living organisms according to their nutritional needs and the sources of energy, hydrogen (electrons) and carbon used (modified from [17]).

Denomination	Energy Source	Hydrogen (e^−^) Source	Carbon Source	Organism
Phototrophs	Light			Green plants, cyanobacteria, purple bacteria
Chemotrophs	Oxidation of organic or inorganic substrates			All others
Lithotrophs		Inorganic compounds (H_2_O, NH_3_, H_2_S)		Green plants, cyanobacteria, purple bacteria, nitrifying bacteria, thiobacilli
Organotrophs		Organic compounds		Animals and most microorganisms
Autotrophs			CO_2_ fixation	Green plants, cyanobacteria, purple bacteria, nitrifying bacteria, thiobacilli
Heterotrophs			Assimilation of organic compounds	Animals and most microorganisms
Photolithoauxotrophs	Light	H_2_O	CO_2_ fixation	Green plants, cyanobacteria, purple bacteria *
Chemolithoauxotrophs	Oxidation of inorganic compounds	Inorganic compounds	Generally, CO_2_ fixation	Nitrifying bacteria (NH_3_), Thiobacilli (H_2_S) **
Chemoorganoheterotrophs	Oxidation of organic compounds	Organic compounds	Assimilation of organic compounds	Animals and most microorganisms

* Some purple bacteria are non-oxygenic, and others use reduced compounds as a hydrogen source [18]; ** Other bacteria may oxidize Fe^2+^.

**Table 3:** Standard reduction potentials of some half-reactions important in biochemistry.

Oxidant (Oxidized Form)	Reductant (Reduced Form)	*z*	E′° (V)
Succinate + CO_2_	Oxoglutarate	2	−0.67
Acetate + 3 H^+^	Acetaldehyde + H_2_O	2	−0.60
Ferredoxin oxidized	Ferredoxin reduced	1	−0.43
**2 H^+^**	**H_2_**	**2**	**−0.42**
NAD^+^ + H^+^	NADH	2	−0.32
NADP^+^ + H^+^	NADPH	2	−0.32
Lipoate oxidized + 2 H^+^	Lipoate reduced	2	−0.29
Glutathione oxidized ** + 2 H^+^	Glutathione reduced	2	−0.24
FAD + 2 H^+^	FADH_2_	2	−0.22 *
Acetaldehyde + 2 H^+^	Ethanol	2	−0.20
Pyruvate + H^+^	Lactate	2	−0.19
Fumarate + 2 H^+^	Succinate	2	0.03
Ubiquinone (Q) + 2 H^+^	Ubiquinol (QH_2_)	2	0.06
Cytochrome b (Fe^3+^)	Cytochrome b (Fe^2+^)	2	0.07
Dehydroascorbate + 2 H^+^	Ascorbate	2	0.08
Cytochrome c (Fe^3+^)	Cytochrome c (Fe^2+^)	1	0.22
Fe^3+^	Fe^2+^	1	0.77
1/2 O_2_ + 2 H^+^	H_2_O	2	0.82
P680^+^	P680	1	1.17

E′° corresponds to the standard reduction potential (pH 7, 25 °C) and *z* is the number of electrons transferred. By convention, the partial reactions are written in the sense of the reduction (oxidant + e^−^ → reductant). (According to [23].) * Free coenzyme, ** For [GSH] + [GSSG] = 10 mM [14].

## Data Availability

Not applicable.

## Abbreviations

CYSS	Cystine
ER	Endoplasmic reticulum
GSH	Reduced glutathione
GSSG	Oxidized glutathione
PPS	Pentose phosphate shunt
ROS	Reactive Oxygen Species
TCA	Tricarboxylic Acid Cycle
